# Supplementary figures and images for: Fitness-Conditional Genes for Soil Adaptation in the Bioaugmentation Agent Pseudomonas veronii 1YdBTEX2
Source: mSystems. 2023 Feb 14;8(2):e01174-22. doi: 10.1128/msystems.01174-22 (PMC10134887; doi:10.1128/msystems.01174-22)

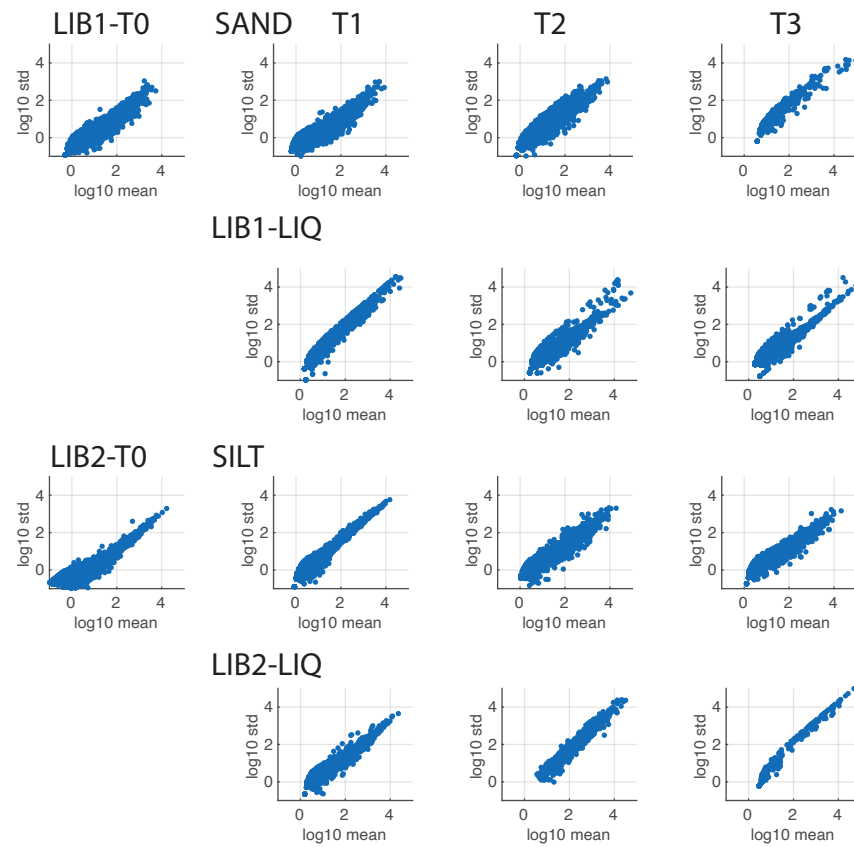

Supplement: FIG S1 [file msystems.01174-22-s0007.pdf]
