# Supplementary material for: Fitness-Conditional Genes for Soil Adaptation in the Bioaugmentation Agent Pseudomonas veronii 1YdBTEX2
Source: mSystems. 2023 Feb 14;8(2):e01174-22. doi: 10.1128/msystems.01174-22 (PMC10134887; doi:10.1128/msystems.01174-22)
Supplement: FIG S2 [file msystems.01174-22-s0008.pdf]

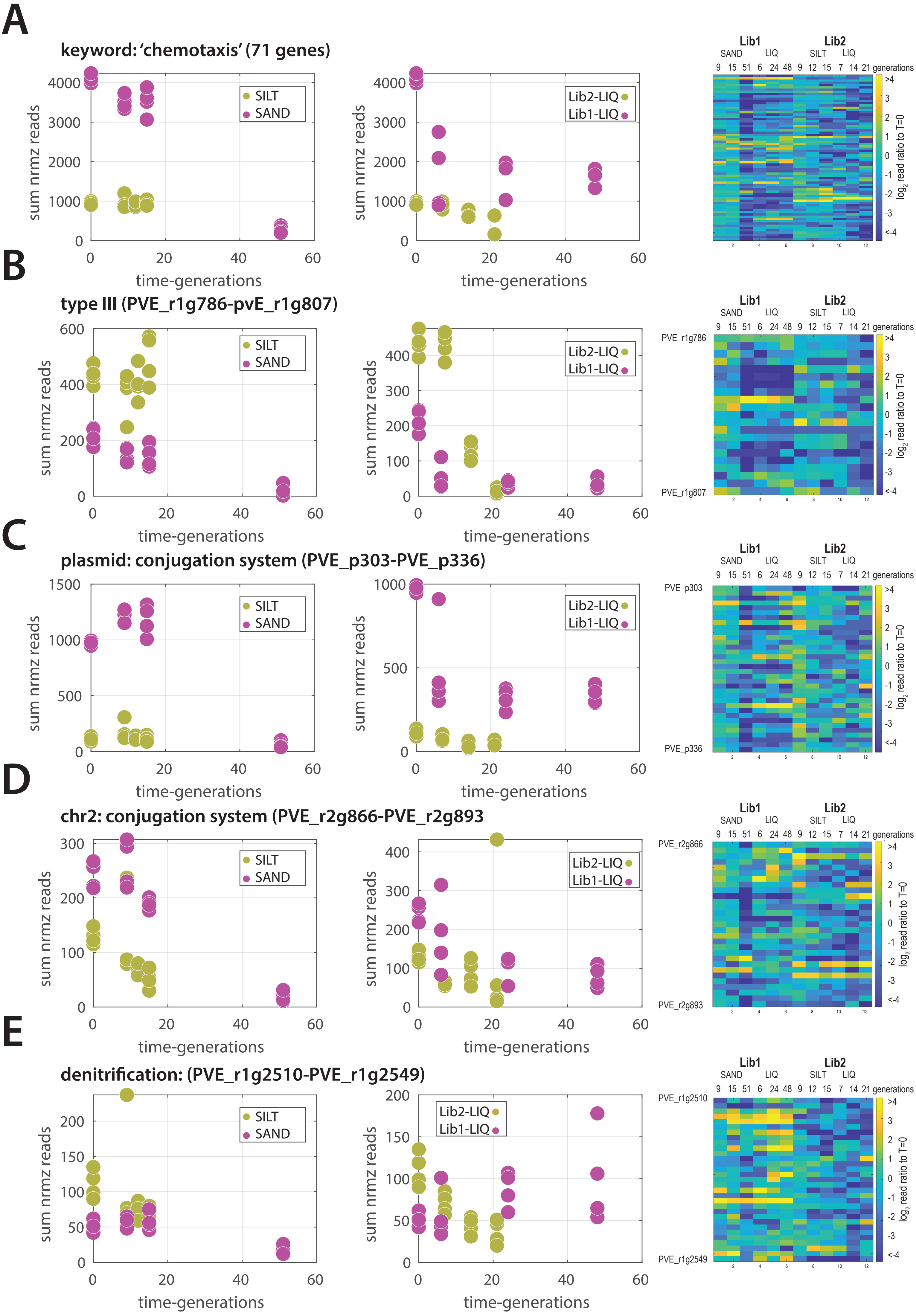
A
keyword: ‘chemotaxis’ (71 genes)
sum nrmz reads
time-generations
SILT
SAND
Lib2-LIQ
Lib1-LIQ
Lib1
Lib2
SAND
LIQ
SILT
LIQ
9 15 51 6 24 48 9 12 15 7 14 21 generations
log2 read ratio to T=0
B
type III (PVE_r1g786-pvE_r1g807)
PVE_r1g786
PVE_r1g807
C
plasmid: conjugation system (PVE_p303-PVE_p336)
PVE_p303
PVE_p336
D
chr2: conjugation system (PVE_r2g866-PVE_r2g893
PVE_r2g866
PVE_r2g893
E
denitrification: (PVE_r1g2510-PVE_r1g2549)
PVE_r1g2510
PVE_r1g2549
